# Adapting a quality improvement collaborative to a new national context: a co-design and feasibility study to improve dementia care in Ireland

**DOI:** 10.1186/s12913-023-10019-3

**Published:** 2023-10-04

**Authors:** Michael Sykes, Lauren O’Mahony, Daisy Wiggin, Suzanne Timmons

**Affiliations:** 1 University College, Cork, Ireland; 2 Northumbria University, Newcastle upon Tyne, UK United Kingdom; 3 Mercy University Hospital, Cork, Ireland

**Keywords:** Adaptation, Audit and feedback, Quality improvement collaborative, Co-design, Feasibility

## Abstract

**Background:**

Adaptation seeks to increase intervention fit with context, an important influence upon implementation. People with dementia in acute hospitals in Ireland do not routinely receive best care. To improve care in Ireland, we sought to adapt an existing quality improvement collaborative, to support the improvement capabilities of recipients of feedback from the Irish National Audit of Dementia.

**Methods:**

The study followed a staged process to co-design adaptations to reflect contextual differences between the English and the Irish healthcare systems, and to explore feasibility of the adapted Quality Improvement Collaborative in Ireland. We used co-design group meetings involving dementia clinicians from three hospitals, delivered the intervention virtually and interviewed healthcare workers from seven hospitals to adapt and explore the fidelity, affective response and reported appropriateness of the intervention.

**Results:**

The intervention required adaptation to reflect differences in strategic intention, ways of working and hospital social structures. There was evidence that the adapted intervention generated a positive affective response, was perceived as appropriate and led to fidelity of receipt and response.

**Conclusion:**

We describe implications for the adaptation of interventions to increase participants’ quality improvement capabilities and highlight the importance of socio-adaptive work. We propose further work to explore antecedents of senior positional leader engagement, to describe the delivery of intra-participant behaviour change techniques and to adapt the intervention to other clinical domains.

**Supplementary Information:**

The online version contains supplementary material available at 10.1186/s12913-023-10019-3.

## Contributions to the literature

- Journals describe interventions to improve care that provide the opportunity to translate learning to new settings.
- We used co-design methods and interviews to adapt and explore implementation of an intervention to increase the quality improvement capabilities of national audit recipients.
- We describe both anticipated and unanticipated benefits from adapting an existing intervention, and highlight the importance of addressing differences in engagement, organisational structure, ways of working and terminology.

## Background

Intervention fit with context is an important influence upon implementation [1]. Adaptation is the intentional refinement of an intervention to increase contextual fit [2]. Methods to adapt interventions to increase fit sit within a staged process involving assessing the rationale, planning and undertaking adaptations, exploration of feasibility prior to delivery at scale [2, 3]. There is a need for empirical studies that extend understanding of the change processes within adaptation [4]. The current paper describes work to adapt an existing intervention to improve the care for people with dementia in England to increase fit with the Irish national context.

Dementia affects over 64,000 people in Ireland [5], and approximately 29% of public hospital patients aged over 70 have dementia [6]. People with dementia do not routinely receive best care in Irish acute hospitals. For example, 94% of case notes do not include collateral information from carers, 81% of patients with dementia are not screened for delirium and 60% do not receive cognitive testing in hospital [7]. Improving hospital care for people with dementia is a policy priority [8].

Audit and feedback (A&F) is a commonly-used intervention to increase the use of evidence-based care [9]. For example, there are approximately 60 national audits in England [10] and about 16 in Ireland [11]. A&F involves gathering data and providing feedback about the quality of care over a specific period of time. There is evidence that the implementation of A&F varies in content, delivery and outcome. A systematic review [9] found that A&F led to a 4.3% median absolute improvement in the delivery of clinical behaviours but had the potential to have a larger effect (interquartile range 0.5 to 16%). Components associated with greater improvement were identified, for example, targeting care behaviours with a low baseline and providing both verbal and written feedback. In addition, theory-informed hypotheses for enhancing A&F have been suggested, for example, delivering feedback to people with greater quality improvement capabilities [12].

The Irish National Audit of Dementia (INAD) collected data in 2019, and provided feedback to the 87% of eligible hospitals that participated in the audit [7]. It involves data being collected manually from hospital records, as well as an environmental walk-around, and interviews with senior hospital staff. The results are fed back at a hospital, regional (Hospital Group) and national level in the form of written reports, aiming to stimulate quality improvement initiatives in response to results and the INAD recommendations.

Work in England sought to enhance improvement from the English National Audit of Dementia (ENAD) by supporting the quality improvement capabilities of hospital level dementia and clinical governance leads in response to audit data [13, 14]. The intervention was a form of Quality Improvement Collaborative (QIC), as described in Table 1.

**Table 1:** Intervention (QIC) content and delivery prior to co-design work to translate to the Irish context [10]

Brief name: Quality Improvement Collaborative (QIC)
Why: To support the quality improvement capabilities of recipients of feedback from the national audit of dementia
What procedures: To develop and implement improvement actions in response to the national audit of dementia through a structured process involving: Analysing performance and specifying target for improvement; Investigating barriers; Identifying actions; Linking performance to priorities; selecting comparators; addressing trust and credibility; reflecting existing workstreams.
What materials: Powerpoint slides and Microsoft Word worksheets
Who provided: Facilitator
To whom: Clinical leads and clinical governance leads from participating hospitals
How: Face-to-face workshop and monthly multisite facilitated telephone calls
Where: Geographically convenient training venue
When: After initial audit feedback. Six-hour workshop and monthly calls (*n*=12)
Tailoring: Undertaken through supporting participants to tailor their response to local circumstances.

It may be more efficient and effective to adapt an existing intervention to a new context, rather than developing a new intervention [2]. Different steps to adaptation have been described, including to: select the intervention that best matches the context, explore the validity of the conceptual framework and the essential components, revise the intervention, and complete a feasibility study of the adapted intervention [3].

The current study sought to adapt the ENAD Quality Improvement Collaborative intervention to Ireland by.

- Working with a co-design group to adapt the intervention to reflect contextual differences between the English and the Irish healthcare system.
- Exploring fidelity, affective response and reported appropriateness of the adapted Quality Improvement Collaborative in Ireland.

## Method

### Our research question was

How should a National Audit Quality Improvement Collaborative be adapted to fit the Irish healthcare context? We sought to answer this question through two work packages.

### Design

**Fig. 1:**
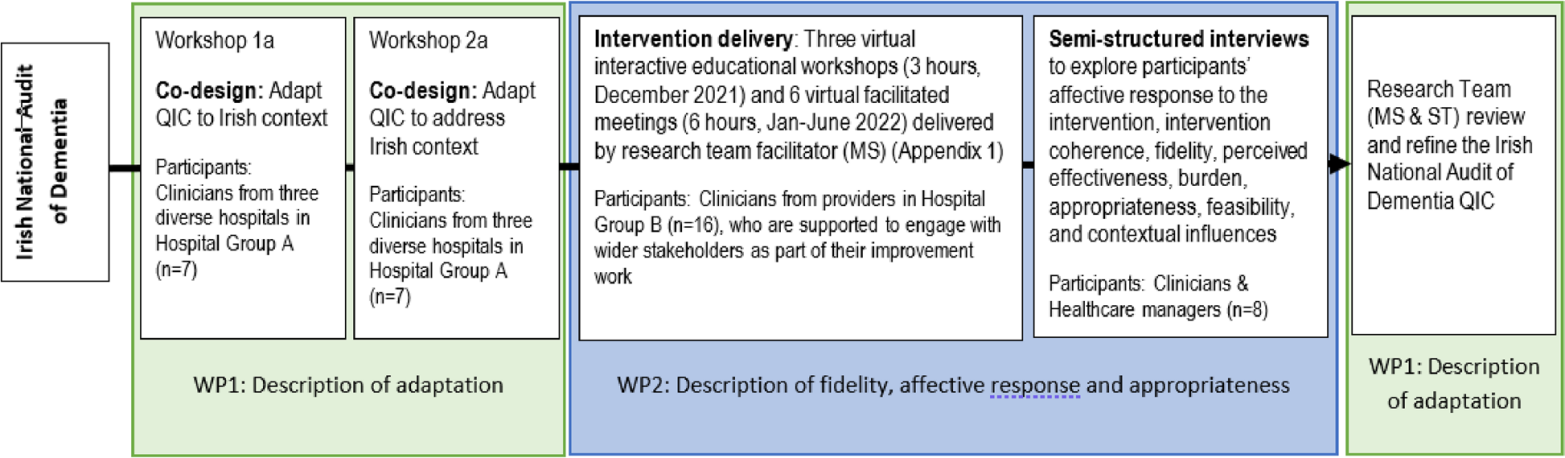
Study design

Qualitative methods are valuable during the adaptation of interventions [3]. This study used co-design and interviews, as illustrated in Fig. 1.

### Work package 1

To adapt the intervention to reflect contextual differences between the English and the Irish healthcare system.

#### Method

Facilitated virtual co-design group meetings. Guidance highlights the importance of identifying and involving members of the target population in intervention development (e.g. [15]). Within the current study, the rationale for this method was to engage potential participants in the refinement of the intervention to support appropriateness of design [16] and hence implementation of the intervention. Using virtual methods for the co-design work was anticipated both to be acceptable and to provide information about subsequent virtual intervention delivery. Iterative meetings with the co-design participant group creates the opportunity for reflective sense-making and discussion that was anticipated to strengthen refinement.

#### Participants

We sought participants who were representative of the target audience for the intervention. These were clinical dementia leads from different clinical professions in three diverse hospitals within one Irish Hospital Group. A list of potential participants was provided by the Hospital Group director of nursing. The potential participants were dementia specialists (senior nurse, allied health professional or consultant) from diverse hospitals. Potential participants were sent a participant information sheet and asked to consider willingness to consent to participation. In Ireland, these hospitals form part of multi-hospital groups, with between 5 and 12 hospitals in a Hospital Group.

#### Co-design meetings

Two virtual co-design meetings were facilitated by MS, who had led the development of the QIC intervention in the English context. The co-design meetings did not seek consensus, but rather wanted to explore how different stakeholders perceived the content and delivery of the intervention.

In the first workshop, the aim of the study (to adapt an intervention developed in England to the Irish context) was discussed and the proposed intervention development method outlined. Participants were presented with a description of the method and results from earlier work in six English hospitals to describe and enhance the ENAD through the delivery of a QIC [9, 10]. This was presented in sections, with each followed by discussion about similarities and differences from what happened at their hospital with the INAD results.

During the second workshop, an overview of the QIC intervention developed in England (Table 1; Additional file 1, Appendix 1) was presented so as to allow discussion about the content, rationale and contextual differences. Participants were presented with the intervention materials (Slides, presenter notes and participant exercises) and asked to consider changes needed to the workshop to reflect the Irish context.

We audio-recorded the workshops and took notes by annotating the intervention materials. Analysis involved listening to the recordings to capture extracts that described whether, how and why to retain and/or change the intervention content and delivery. We also classified the proposed adaptations [17].

### Work package 2

To explore fidelity, affective response and reported appropriateness of the adapted QIC.

#### Design

Adaptations may have a beneficial, detrimental or neutral affect upon implementation [17]. To assess this, we undertook a feasibility study involving intervention delivery and participant interviews.

#### Site

We recruited eight hospitals from a second Hospital Group in Ireland.

#### Participants

A list of potential participants was provided by the Hospital Group director of nursing. The potential participants were the lead(s) with responsibility for developing the hospital response to the audit results (typically Dementia Nurse Specialist/Advanced Nurse Practitioner and/or the medical consultant lead for dementia) and others involved in the Hospital Group’s response to the audit. Potential participants were sent a participant information sheet and asked to consider willingness to consent to participation. As for Work Package 1, the sample included Model 2, 3 and 4 hospitals from both rural and urban settings.

#### Intervention

An initial interactive virtual workshop (Additional file 1, Appendix 1 and 2) was delivered over three one-hour sessions, followed by six facilitated once-monthly meetings. The meetings sought to provide the physical and social opportunity for participants to collaborate, and the motivation and capability to undertake improvement actions in response to the INAD results and their local circumstances.

Up to 7 weeks after the sixth facilitated meeting, virtual semi-structured interviews (Mean duration 45 min) explored the following implementation outcomes: participants’ affective response to the intervention, intervention coherence, fidelity, appropriateness (perceived fit), feasibility (suitable for use) and contextual influences [16, 18]. Participants were sampled purposively for diversity of profession and employing organisation. The topic guide is presented in Additional File 1, Appendix 3. The interviews were audio recorded and transcribed verbatim. Interpretation sought to identify implications for both adapted and retained content.

**Fig. 2:**
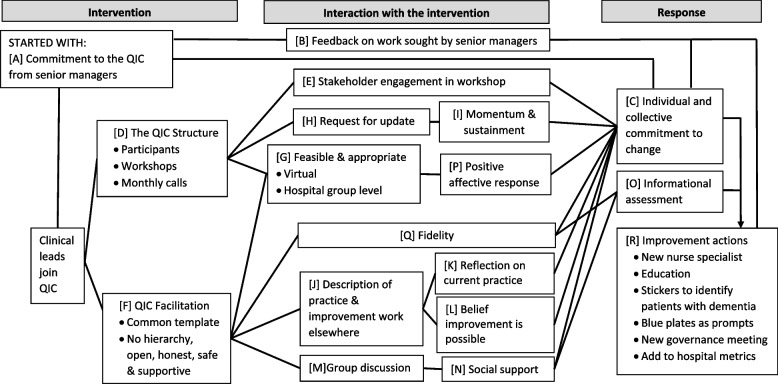
Model describing participants’ reported experience of the intervention, their interaction with the intervention and their response

Analysis and synthesis: Interview transcripts were analysed using framework analysis [19]. Analysis involved familiarisation with the data through reading and re-reading the transcripts and listening to the audio-recordings and making notes. Data were managed by inductively developing an initial thematic framework from the transcripts. Following familiarisation with 8 transcripts, an initial framework (See Supplementary materials) was developed by MS and sent to LOM for challenge. Challenge considered distinction between categories, gaps, clarity, levels within the data and separation of the work of the QIC and the work being done by participants. We indexed and sorted data using the revised framework (See Supplementary Materials), which evolved further as new themes were identified and previous ones amended and/or combined. Data summaries presented alongside source quotes and proposed exemplar quotes were produced (over 100 pages) by MS, reviewed by LOM and amended through discussion. The proposed understanding (Fig. 2) and exemplar quotes were discussed until a common understanding developed. Abstraction, interpretation and synthesis involved describing and linking categories, before drawing upon wider literature in order to provide an explanation of the data.

## Results

### Work package 1

Senior clinicians (consultant geriatricians, advanced nurse practitioners, occupational therapists; *n* = 7) from three hospitals took part in the first co-design meeting. The same clinicians also took part in the second co-design meeting, although this was split into two groups, meeting on different occasions, as not all could attend a single occasion. In total, the co-design meetings lasted 4 h and 50 min.

Table 2 presents the findings from the co-design group, and the subsequent adaptations suggested for the QIC in an Irish context. Additional file 1, Appendix 4 extends Table 2 by providing exemplar quotes and by applying Stirman and colleagues’ [17] classification to the adaptations.

**Table 2:** A summary of the feedback from the co-design group and the subsequent adaptation

Summary of co-design group feedback	Adaptation
**Engagement, reach and structure** 1a. There may be a lack of positional leader engagement with the audit (INAD) findings, and/or work to improve the care for people with dementia. This lack of engagement may reduce both participants’ and stakeholders’ willingness to commit time to the QIC 1b. Senior positional leaders may not prioritise INAD results. This might reduce organisational commitment for change. Linking INAD to other priorities including complaints, incidents, reputation, length of stay and/or other costs may increase positional leader engagement and organisational commitment 1c. It is important to engage clinical staff in order to gain buy-in for change 1d. The INAD report, and dementia care more broadly, may not be discussed at committees. These structural differences need to be reflected in the work to develop commitment	National Dementia Office to engage senior leaders in the QIC Content within the QIC that seeks to support participants’ work to engage stakeholders to move earlier, so that it is considered before exploring influences upon performance, which requires stakeholder engagement. Stakeholder engagement to be revisited in Workshop 2 by adding examples of the different stakeholders to engage, using ‘influences’ exercise both to engage stakeholders and gain perspectives upon influences, and again in Workshop 3 (here adding discussion of impacts from INAD performance that might relate to local priorities e.g. patient outcomes, costs, length of stay) Add a new engagement activity, so that participants consider the influence and interest of different stakeholders Remove *committee* sense-making from logic model and identify other ways to engage stakeholders
1e. If the people leading hospital improvement from INAD knew about existing actions, they may be able to amend them to improve INAD performance, but often they would not know about them 1f. There may be challenges with gaining organisational commitment due to a lack of clear reporting lines 1 g. There was the potential for gaps in communicating agreed improvement actions, and that engaging stakeholders may be more relational than structural	Add post-workshop work to consider: • stakeholders’ improvement goal(s) • stakeholders’ understanding of performance in INAD • strategies and workstreams related to the identified actions • local internal communication arrangements
**Fit with working patterns and terminology** 2a. Shorter sessions may fit better with participants’ working patterns	The workshops should be split into three one-hour sessions. Reduce workshop duration by moving exercises outside the workshops and replacing them with group practice exercises (e.g. After workshop 1: Discuss INAD priorities with your colleagues. What does meeting these standards involve? Who are the stakeholders?) To assist with sense-making, add a reminder of previous content at the beginning of each workshop
2b. Virtual delivery would be appropriate	Workshops are now often delivered virtually due to Covid-19 Pandemic and would reduce the relative travel time costs from splitting workshop into three sessions
2c. Differences in terminology in Ireland compared to England. Using Irish terminology would support understanding and credibility	Check terms used throughout
**The actors involved** 3a. The clinical governance roles present within English hospitals that were studied, were not present in Ireland. These structural differences need to be reflected in the informational appraisal work	Change participants so as not to seek ‘clinical governance leaders’ for the workshops
3b. Participants’ reported a preference to collaborate with other hospitals within their Hospital Group. This was anticipated to create social opportunity to collaborate with hospitals that were similar, providing the opportunity for knowledge translation Working with hospitals within a Hospital Group was also anticipated to increase positional leader support and organisational commitment for change	Limit the QIC to one Hospital Group
**Tailoring** 4a. Participants currently select priorities for improvement based upon ease of action rather than impact upon meaningful outcomes. This may result in the selection of less effective actions, undermining the ability of the intervention to improve care	To enable collective discussion of priorities with the greatest opportunity to improve patient outcomes, present INAD data for all participating sites, allow two minutes reflection, then ask what they would celebrate and what might be their priorities for improvement
4b. There were differences of opinion about who would undertake a care practice, where and when. This reinforced the need to include content that supported teams to describe local practice before considering influences upon the performance of this practice	To develop collective understanding of how to specify the target for improvement, add discussion to specify target for improvement using an example from the INAD standards (e.g. What does meeting the delirium screening standard involve, who does it, where, when and with what materials) as a step prior to exploring influences in workshop 2
4c. Participants reported selecting improvement actions from a small range of potential implementation strategies	Add example of a completed logic model and group work to consider influences upon one target from the audit and potential actions Target for improvement used in the example to be selected based upon discussion in Workshop 1
4d. There was the potential for gaps in communicating agreed improvement actions	Add content about developing an action plan Revisit stakeholder analysis to include consideration of stakeholders to the actions

Through the co-design work we found that the Quality Improvement Collaborative needed to be adapted to reflect contextual differences. More specifically, the adapted intervention sought to influence strategic intentions, fit with ways of working and reflect differences in the social structure within Irish hospitals.

Contextual differences shaped how the intervention sought to influence strategic intentions [20]: The co-design group identified that there may be a lack of positional leader engagement with both INAD and the QIC and that this might impact upon both the provision of organisational resources and clinical staff’s buy-in and willingness to commit time to the work of the QIC. To address this, the co-design group proposed changing the source of the QIC, so that the positional leaders are invited to participate by the National Dementia Office. Once engagement with the QIC had been supported, the group proposed extending the work within the QIC to develop organisational commitment through positional leader engagement by linking INAD to other priorities including complaints, incidents, reputation, length of stay and/or other costs. Working with hospitals within a Hospital Group was also anticipated to increase positional leader support and organisational commitment for change.

Contextual differences affected the fit between the QIC and existing ways of working: The co-design group identified that short sessions may fit better with participants working patterns and that virtual delivery would be appropriate. The co-design group reported that differences in terminology between Ireland and England needed to be reflected within the intervention content to support participant understanding and their perceptions of intervention appropriateness and facilitator credibility. Clinical governance roles present within English hospitals were not present in Ireland, this stimulated changes to the QIC participants and extension of the work to implement stakeholder engagement in the informational appraisal.

The QIC supports teams to tailor actions to influences upon clinical practice. Participants described differences in the enactment of these care practices which stimulated minor adaptations to the tailoring content. Similarly, participants described the need to adapt the QIC to support the selection of improvement actions: Participants described currently selecting priorities for improvement based upon ease of action rather than impact upon meaningful outcomes. This may result in the selection of less effective actions, undermining the ability of the intervention to improve care.

Existing social structures may reduce the impact of the QIC: Working with hospitals within a Hospital Group was anticipated to create social opportunity to collaborate with hospitals that were perceived as similar, and that this would support knowledge translation. The co-design group reported that current communication structures may not provide participants with information about existing improvement work and that this may inhibit participants’ ability to bring these resources to bear upon dementia care. Similarly, existing communication structures may inhibit the clinical leads’ ability to engage stakeholders in improvement actions. To address this, we adapted the intervention to incorporate additional stakeholder engagement work.

### Work package 2

During Work package 2, the intervention, described in Appendices 1 and 2, was delivered to 16 healthcare workers from 8 hospitals. The QIC participants were: senior clinicians (e.g. geriatricians, dementia nurse specialists, senior occupational therapists) from eight hospitals in a different Hospital Group from the one involved in the co-design work (*n* = 14); a Hospital Group-level project manager; and a senior third-sector community resource worker. The 16 healthcare workers were involved in the workshops and facilitated virtual meetings.

Eight QIC participants then participated in virtual semi-structured interviews to explore the fidelity, affective response to, and reported appropriateness of the intervention. The participants were from seven hospitals and a Hospital Group-level project manager. A potential participant from the eighth hospital agreed to take part but withdrew shortly before the interview due to a Covid outbreak.

Table 3 summarises the findings. Figure 2 illustrate our synthesis of the findings, describing the implementation of the adapted Quality Improvement Collaborative, and participants’ subsequent interaction with the intervention and their later response.

*We found that the new approach to gaining* **commitment to the QIC from senior managers*** led to participant commitment, and led the senior managers to seek feedback on progress which further increased participant commitment:*

**Table 3:** A summary of findings from work Package 2

In summary, we found:
• the new approach to gaining commitment to the QIC from senior managers led to participant commitment, and led the senior managers to seek feedback on progress which further increased participant commitment
• support for the adapted QIC Structure, but that additional participants may be beneficial
• support for the retained approach to QIC Facilitation
• participants undertook sense-making work about the QIC. The outcome of this assessment was that it was feasible and appropriate
• participants supported retained content, particularly the description of practice from elsewhere, group discussion and the request for updates as important content within the QIC
• the description of practice elsewhere, reflection, belief improvement is possible and social support helped both the informational assessment and the commitment to change
• there was a positive affective response to participation in the QIC
• evidence of fidelity of receipt and response. Further work may be needed to achieve fidelity of enactment of linking the service improvement work to existing workstreams
• participants described undertaking a range of improvement actions, also known as implementation strategies. These included seeking new funding, education, prompts, and audit and feedback

As determined through Work Package 1, to support engagement, the National Dementia Office approached the Hospital Group Director of Nursing and asked for permission to deliver the QIC, and to help identify clinical leaders from within their hospital group who might be potential participants. Participating clinical leaders (e.g. consultant geriatrician, advanced nurse practitioners) described this as a sign of **senior leadership commitment** for the work (Fig. 2A).

“What I felt from this [the QIC] was, because it came from the very top, the hospital manager asked me to be the link for this hospital in the group, I felt it was more of a buy-in from hospital management and feeding back to them and I think that was the difference.” (Participant 3)

Senior leaders were reported to have bought in to the QIC and subsequently [B] **sought feedback** about the work being undertaken through the QIC.

“Another thing that's come out of it is, Umm. Well suppose from organizational commitment is like we do have the buy in from your Directors of Nursing and we do, and it is fed back. So, I'll go to the Director of Nursing meeting in the group and I'll feedback any of the, I suppose, just feedback on how we're getting on with the project. So they were aware of what's actually happening on the ground.” (Participant 2)

Anticipation of monitoring by positional leaders impacted upon [C] commitment by supporting participants’ enrolment:

“When people know that your results are being talked about to other hospital groups, you kind of, I suppose, do want to be doing better.” (Participant 6)

*We found that support for the adapted QIC Structure, but that additional participants may be beneficial:*

Work package 1 adapted [D] the** structure of the QIC**. The number and spacing of the **workshops** and **monthly calls** was important to the subsequent QIC participants. This change to fit with ways of working also provided the unanticipated opportunity for reflective sensemaking:

“it probably would have been nice to have one workshop or maybe 2. But …. say you did all your workshops in one day. I don't think that would have been right either. I think because there was a lot in the workshops and we didn't have time for reflection between workshops and I think reflection is huge… I think it was more valuable because when we spoke you had time to reflect on it… However, I think the monthly meetings. Yes, they're perfect but I do think it would have been valuable to meet in person as well…Maybe, maybe biannually or quarterly.” (Participant 3)

We adapted the content of the QIC to provide further support for stakeholder engagement. Work package 2 identified the opportunity to extend this by identifying the correct participants to be involved in the workshop. Participants highlighted that this may be associated with [E] **stakeholder engagement** and [C] subsequent **commitment to change**:

“Might have brought somebody different onto the group. Maybe if I was back again, I might have brought the CNM [Clinical Nurse Manager] from the specialist geriatric ward because it would have been a learning curve for her in hindsight, I suppose.” … “maybe, I think we were vague in the beginning, but that could have been our own fault. Maybe we weren't engaged enough to start off with” (Participant 4)

*We found support for the retained approach to [F]* ***QIC Facilitation***:

Work Package 2 participants identified elements not in need of adaptation. They reported that the facilitation of the QIC was important, and that both the Theoretical Domains Framework [21]** template** and input from other participants was useful to developing actions.

“I suppose just talking with the facilitator [was most useful for developing action]. And then he gave us, like, he would have given us kind of the sort of template [the Theoretical Domains Framework] I suppose or like kind of the, like, instructions or the headings, just to like get us thinking about like the barriers. Maybe, how we can overcome it and that sort of thing. And then as well as that, hearing about other teams who were involved in the workshop and hearing about their success stories.” (Participant 1)

Participants’ assessment that the intervention should be part of their work was influenced by the **lack of hierarchy**, and the **open, honest, safe and supportive** nature of the collaborative, that was generated through facilitation:

“it's good from that perspective that you know it's open, it's honest, there's no hierarchy and everybody, everybody is included … That is reassuring when you're sort of struggling to get things moving that other people, you know, other disciplines will come on and say, “we haven't made progress because…” and they'll tell you why. … I don't think there was anybody making anything up, and ‘look at us and we're wonderful and we're the best in the group’, there's none of that, you know, it is, it is, it's truthful, it's honest and it's open and everybody is willing to sort of share ideas like they're there for sharing.” (Participant 5)

“give everybody the opportunity to voice their opinion and [facilitator] was also very supportive of places that felt that they might be a little bit behind or didn't think that they were making as much progress.” (Participant 4)

*We found that participants undertook sense-making work about the QIC. The outcome of this assessment was that it was [G]* ***feasible and appropriate***

“And look, you're going to be sceptical in the beginning. You're not going to know. You don't know what's going to be asked of you. And is it going to be another another, something that's going to create a whole lot of work, but it couldn't be anything further from the truth. I actually look forward to the Thursdays [the monthly calls] actually for the hour.” (Participant 4)

Interviewer: “Did you feel like that it [the QIC] fit in with your ways of working?

Participant 1: “I did, yeah. And I actually thought it was good. Like it was a realistic, I think how they did like an hour, once a month. I think, you know, if we do it now every week or, you know, if you are as in the month, we'd be struggling to kind of to do it whereas an hour a month is realistic like, you know.”

Most Work package 2 participants had a positive opinion about the **virtual delivery** of the QIC, a few described that it may be useful to have some face-to-face meetings:

“the fact that it's done virtually as well as it's helpful. It seems you know, it's the way things are are moving.” (Participant 1)

“Personally I don't particularly like educating over a computer or zoom…As far as I'm concerned, I don't think they all should be face to face because I think that will be unachievable and I think people get you know it’s, it's hard to get out, It's hard to even get the hour out of your work time. I don't think it's, I don't think that's necessary. I don't. I think there should be a mixture of it certainly.” (Participant 3)

There were indications that being virtual helped with stakeholder engagement. A reliable internet connection was important to participants:

“Yeah, uhm, I found it beneficial uhm 'cause you know, you could just nip into your office, you know I'm lucky, I have my own office, but I share it with one other colleague. I have a camera. So it was easy for me.” (Participant 6)

The QIC was adapted so as to be delivered to participants from one Hospital Group. Participants reported that working with others within their **Hospital Group** was appropriate, but that there may be some benefits from widening it to others beyond the group, if those hospitals were viewed as similar:

“I think we have more of a link with our hospitals in our own areas. So, and we would probably know each other in our own areas in our own hospital groups, so maybe it wouldn't, but I think it is nice to, because we we're a regional hospital here so we get patients from all of the other hospitals we're involved with, so It's nice to link up which hospitals of our same size and smaller and bigger. No, I think it was nice to have the regional to be honest with you.” (Participant 4)

Interviewer: How would you feel if it wasn't the group, but if it was 32 from across Ireland. How would you feel about that?

Participant 7: “No, I prefer what we’re doing at the moment. Now I do think sometimes, I do think it would be nice to see [a different Hospital Group] because I feel they would be more in line with [our Hospital Group]. If you were talking then about [Hospital Group] and [Hospital Group], like they're more highly populated, their services will be more advanced. But, … [a Hospital Group with a] similar in catchment area, I would be interested in seeing where are they going? How are they getting on? What have they done? What can we take from them if you know what I mean?”

*We found that participants supported retained content, particularly the description of practice from elsewhere, group discussion and the request for updates as important content within the QIC:*

The monthly calls asked participants to provide an update on the actions to improve. The [H] **request for update** was reported as something that helped participants to keep on track and sustain [I] **momentum**.

“I suppose coming back every month that people are feeding back what they’re doing. So it kind of helps to keep the momentum going. I think if the collaborative wasn’t there, the momentum wouldn’t have been there.” (Participant 2)

Continuing to provide updates, beyond the funded intervention delivery, might help sustain the improvement:

“I would like that the future that this continues and maybe, doing a report after year or whatever, and how far we got and where we’re going and and to meet and just to continue on and you know, just because you get something over the line doesn’t mean it stops there. You know, I mean, we’ve often got stuff over the line and next thing something happens and it’s gone. I think to continue to make people focus on our objectives, and if not, something else will come in our way and then maybe it’s the focus will go off of it.” ’Participant 3)

Hearing a [J] **description of practice and improvement work elsewhere** and [K] **reflecting on current practice** can both create discomfort and [L] **belief improvement is possible**, impacting on [C] **commitment to change**:

“I think even just sharing the problem with the others kind of helped to think, ‘Oh yeah actually this is what I can do’, instead of, ‘I’ve hit a brick wall, now I have to stop’, you know. So it definitely helps.” (Participant 2)

Participant 7: “I remember coming into one of your meetings. And I came away very demoralised, yeah? Yeah, I said, ‘God, I’m going nowhere, I just can’t seem to implement change’. Yeah, I came, yeah, it can be, yeah, it can be demoralising alright yeah.”

Interviewer: “And what was the impact of that upon what you then did?”

Participant 7: “I suppose I actually went away, listened to my peers from the different hospitals and went back, how can I bring what they are talking about back to what I’m trying to do, you know.”

[M] **Group discussion** by participants in the collaborative provided [N] **social support** through feedback and practical ideas:

“I actually thought they [the facilitated meetings] were good because I suppose it, you know, usually you work in isolation in a lot of projects of different hospitals, and this, I really found, for one, there was two sides to it that it made you realise what you were doing good in in the site here. Sometimes you think you're not doing, you panic and you say ‘’Oh my God, we're so far behind on things’, but when you hear what's going on in other hospitals and, you know, I know that every area is different. But you're you definitely pick up something from every interaction. And you'll hear different ideas and approaches. So I found that good.” (Participant 8)

*We found that the description of practice elsewhere, reflection, belief improvement is possible and social support helped both the [O] ****informational assessment*** and [C] ***the commitment to change***:

“One of the girls said, ‘but you have done great work, you're getting every single patient when they come into the hospital, they're all being assessed, you know, so every single patient that comes in is being assessed already. So you've actually done great work, you know’. And I think even just working with each other, you know, getting the support from your colleagues in other hospitals say, look, you know, focus on one area and then just, you know, make it smaller, bring it in somewhere else or maybe just park it, leave it for a month and then come back and, you know, bring it in with another change, or, I think just even I suppose it's like it's a problem shared is a problem halved I think sometimes as part of this group.” (Participant 2)

“I sold it a little bit. Just to, maybe, manipulate how I was getting from A to B, in the best interest of the project and getting it up and running. You know, I used what other people were doing to say, ‘this is happening lads, it's happening in the [hospital group], we're gonna have to start making it happen in here’.” (Participant 5)

*We found that there was a [P]* ***positive affective response*** *to participation in the QIC*

We interpreted this as an indicator of acceptability:

“it actually was pleasant to work on something that wasn't Covid related. I felt it was also great to start thinking about improvements to come.” (Participant 3)

“we've made progress over the last six months and we'd be certainly very happy to either promote it or be involved in the future or we wouldn't have a negative word to say about this personally. So you know, very pleased. Really. Yeah. Yeah.” (Participant 4)

“the other thing I we really enjoyed about it was, uhm, there was a multidisciplinary approach to the meetings. It was not just nursing staff or, it was huge area of expertise from everybody.” (Participant 3)

“I liked the way it worked. It was informal. There was a bit of, you know, there’d be a few giggles and I think that is helpful, you know.” (Participant 5)

*We found evidence of [Q]* ***fidelity*** *of receipt and response. Further work may be needed to achieve fidelity of enactment of linking the service improvement work to existing workstreams:*

“I think that it was asking questions of us about how we deliver care. It was making us uh, investigate and, you know, look and develop an insight into how we were doing it here and where we doing it right or what could we improve on. It was just asking questions of us to to kind of provoke thought around the whole process of delivering dementia care and it asked us, really: What did we need to do to improve? What were the most pertinent uh areas that we needed to improve upon? And it's, you know, forced us to look at those areas. And um, it's like a PDSA kind of a cycle basically and decide how we were going to develop means and ways of making improvements around the areas that were weak. And then we fed back regularly, and you know it forced us on to do that even in times of Covid, it asked those questions of us and we were able to, work on it together and deliver what was required.” (Participant 4)

“I suppose it made us kind of find out, OK, like is it even you know, who's invested in this project? Who are we trying to target for each of these? Uhm, each thing, each aspect of the project that we're trying to implement? Who is it that we're trying to focus on? Who do we need to get to be invested in the project?” (Participant 6)

Participants described both [O] undertaking **an informational appraisal** to identify local priorities, analyse influences upon performance towards that goal and select actions aligned to those influences and [C] the **development of individual and collective commitment** to change consistent with the intended response (Additional File 1, Appendix 1):

The participant-selected target for improvement was initially common across seven sites (to improve the assessment of delirium), but over time this broadened to other targets:

“out of the eight hospitals that are sitting on [the group] and seven of them picked a assessment of delirium and one hospital, as I say, the [hospital] picked environment, but you know, they're all interchanging now, even the [hospital] are working on activity packs and you know they're working on their assessment of delirium as well.” (Participant 2)

*We found that participants described undertaking a range of [R] ****improvement actions****, *also known as implementation strategies. These included seeking new funding, education, prompts, and audit and feedback.

## Discussion

Adapting an intervention to increase fit with a new context may support implementation [1] and make the interventions more likely to achieve the same outcomes than ones that have been simply replicated [22]. Using co-design methods with clinicians from three hospitals, we were able to adapt to the Irish context, an intervention to support the quality improvement capabilities of national audit feedback recipients. We identified the need to adapt the intervention to reflect differences in strategic intention, ways of working and hospital social structures.

We delivered the adapted intervention to clinicians from 8 hospitals in a different Hospital Group. Through interviews we identified support for the adapted approach to engage senior leaders, and to shorten workshops, increase work to support participants’ stakeholder engagement and deliver the QIC virtually. There was also support for retained content, including monthly calls and facilitation that sought to share practice and seek updates. The adapted intervention generated a positive affective response, was perceived as effective and appropriate and led to fidelity of receipt and enactment. Consistent with the idea of core and peripheral components [4], we found that, as within the English context, the intervention supported the commitment and informational assessment of participants to undertake actions to improve performance described in the national audit.

We identified that multiple, short, virtual workshop sessions may fit better with participants working patterns (compatibility; [1]), positively impacting upon participation. An unanticipated benefit from this change was that providing weekly sessions created the opportunity for increased reflection. The reflection work aligned to the target practices in the intervention (an illustration of ‘interactional workability’ [23]), for example, exploration of influences upon performance and undertaking work to identify and engage stakeholders. We highlight three implications from this: Firstly, that the space between workshops might appear to have an absence of content, but there is important work being done, as participants make sense of what is required and start to operationalise it. Describing these gaps between sessions as content within the intervention manual would support later replication. Secondly, that if – as we expect – this reflection work is important, providing additional support for reflection might make the work more efficient and/or effective. Thirdly, creating the opportunity to surface some of these reflections in the subsequent sessions might both enable barriers to the improvement work to be addressed and inform future content of the intervention.

Differences between England and Ireland in organisational governance structures were reported to influence reach and response to the intervention. For example, in terms of logistical fit [24], there were fewer clinical governance staff within the Irish hospital system, as a result we removed the requirement for their involvement in the QIC. In addition, formal governance communication routes were reported as being less important in Ireland than more informal, relational networks. Whilst this might speak to logistical fit with organisational structure, we interpreted this as relating to philosophical fit, that change is made through socio-emotional work [24]. To address this, we increased support for stakeholder engagement. Previous studies have found that the development of effective social relationships that are important for quality improvement work can be difficult and time-consuming work [24]. Our response to address the need for increased support for engagement resonates with Stephens and colleagues’ proposal to prioritise this socio-adaptive work [25]. Consistent with Duggleby et al.’s [3] adaptation steps, there was a need to adapt the terminology used, such as amending reference to ‘safety huddle’ to ‘safety pause’ and removing reference to ‘named nurse’.

We identified the delivery of behaviour change techniques by participants to their peers. The logic model for the intervention (Additional File 1, Appendix 1) describes behaviour change techniques [26] employed by the facilitator to implement specific procedures in the response to national audit data. For example, using *goal setting* to support national audit recipients to identify influences upon practice. In describing their experience in the QIC, participants described that they also acted as a result of input from other participants, this included as a result of *social support* and *monitoring of others with and without feedback* [26]. We recommend that future studies of collaboratives or groups similarly explore the delivery of intra-group active ingredients.

One implication from the delivery of BCTs by participants is that the characteristics of participants may influence whether they deliver these BCTs, thereby impacting upon the effectiveness of the intervention; for example, participants’ capability or motivation to support other participants may influence the delivery of social support. Similarly, the extent to which other participants’ organisations are seen as comparable and/or that have higher performance from which to learn may influence willingness to adopt/adapt a tested improvement action; this resonates with our findings that participants wanted to be in a Collaborative with particular hospitals (i.e. those within the same group or that are similar in size). Careful consideration and selection of both the hospitals and the participants, or the development of additional content to mitigate factors which may inhibit the delivery of BCTs or the sharing of learning, may increase intervention effectiveness.

There are strengths and limitations to this work: We engaged stakeholders from different Hospital Groups, hospitals and clinical professions in order to identify how to adapt the intervention and then to explore the feasibility of the adapted intervention. Prior intervention development was led by MS who iteratively developed and tested the stakeholder-, evidence- and theory-informed intervention, describing the intervention in a detailed logic model [14]. Guidance [3] recommends adaptation is a planned pre-delivery process that begins by selecting the intervention that best matches the new target population and context. The Irish National Audit of Dementia is based upon the English National Audit of Dementia. As such, we selected the intervention that had been developed to support the quality improvement capabilities of national audit feedback recipients in England. It is possible that there were alternative interventions that may have provided a better initial intervention. MS’ involvement and the detailed logic model provided the deep understanding of the prior intervention that is needed for adaptation [2, 3]. However, it is possible that MS’ involvement may have inhibited the co-design group’s willingness to amend the intervention, although Table 2 suggests that they were comfortable to propose Irish differences and adaptations. Similarly, following intervention delivery, MS and LOM undertook interviews with participants. It is possible that social desirability influenced participants to respond positively to MS as QIC facilitator and interviewer; we compared transcripts and did not identify differences in responses which might allude to this influence and have provided extensive quotes to support trustworthiness and verification. We sought to engage potential recipients in the intervention development work. Whilst the ENAD intervention had involved carers of people with dementia in the intervention development work, the subsequent intervention was to be delivered to healthcare workers. As such, we did not include patients or carers in the adaptation. It is possible that including patients and carers in the codesign work may have led to different adaptations, although it might also have impacted upon participant openness [27]. Context changes over time, and as such, there may be a need for further adaptation prior to further intervention delivery [28].

The effectiveness of audit and feedback may be influenced by the quality improvement capabilities of the feedback recipients [8]. Implementation laboratories involving collaboration between national audit providers and research teams offer the potential to undertake sequential effectiveness studies to generate generalisable knowledge [29]. Identifying how to adapt effective interventions to different contexts will help realise the promise of such laboratories; for example, to take account of differences in engagement, communication structures and feedback reach. This paper describes one approach to adapt an audit and feedback co-intervention to a different national context. Parallel work to consider adaption between clinical domains (for example, from adapting from dementia to stroke care) would support national audit providers and commissioners such as the National Centre for Clinical Audit and the National Office of Clinical Audit in Ireland, and the Healthcare Quality Improvement Partnership in England, to realise any benefits at greater scale.

## Conclusion

Adapting an intervention to a new context may support implementation. We adapted an English Quality Improvement Collaborative to the Irish context by: working with a co-design group to refine the intervention to reflect contextual differences in the Irish healthcare system; exploring whether it is feasible, acceptable and appropriate to implement the adapted Quality Improvement Collaborative in Ireland. We found the need to adapt the intervention to reflect differences in strategic intention, ways of working and hospital social structures. There was evidence that the adapted intervention generated a positive affective response, was perceived as appropriate and led to fidelity of receipt and enactment. There was support for introducing the intervention to senior positional leaders in the Hospital Group through the National Dementia Office, and that subsequent senior positional leaders’ engagement was considered important to the success of the collaborative. Participants reported that the description of practice elsewhere was a driver of intention to change, provided practical ideas and helped with their local problem solving, while social support was a key benefit of the collaborative. Future work to adapt the intervention to support the quality improvement capabilities of recipients of feedback from other national audits, may help to increase the effectiveness of national audits at scale.

### Supplementary Information

**Additional file 1:** **Appendix 1.** Intervention logic model. **Appendix 2.** TIDieR [30] and FRAME-IS [31] description of the post-adaptation QIC. **Appendix 3.** Interview topic guides (v30May22). **Appendix 4.** Exemplar quotes from Work package 1.

**Additional file 2:** **Supplementary material.** The evolution of data categories.

## Data Availability

The datasets generated and/or analysed during the current study are not publicly available as they contain contextual information which would enable identification of participants, but extracts with such identifiable information removed are available from the corresponding author on reasonable request.

## Abbreviations

A&F: Audit and feedback
BCT: Behaviour change technique
ENAD: English national audit of dementia
INAD: Irish national audit of dementia
LOM: Lauren O’Mahony
NPT: Normalisation process theory
MS: Michael Sykes
QIC: Quality improvement collaborative
ST: Suzanne Timmons
