# Supplementary material for: Adapting a quality improvement collaborative to a new national context: a co-design and feasibility study to improve dementia care in Ireland
Source: BMC Health Serv Res. 2023 Oct 4;23:1056. doi: 10.1186/s12913-023-10019-3 (PMC10548569; doi:10.1186/s12913-023-10019-3)
Supplement: Supplementary file 1 — Additional file 1: Appendix 1. Intervention logic model. Appendix 2. TIDieR [30] and FRAME-IS [31] description of the post-adaptation QIC. Appendix 3. Interview topic guides (v30May22). Appendix 4. Exemplar quotes from Work package 1. [file 12913_2023_10019_MOESM1_ESM.docx]

**Appendix 1:** Intervention logic model


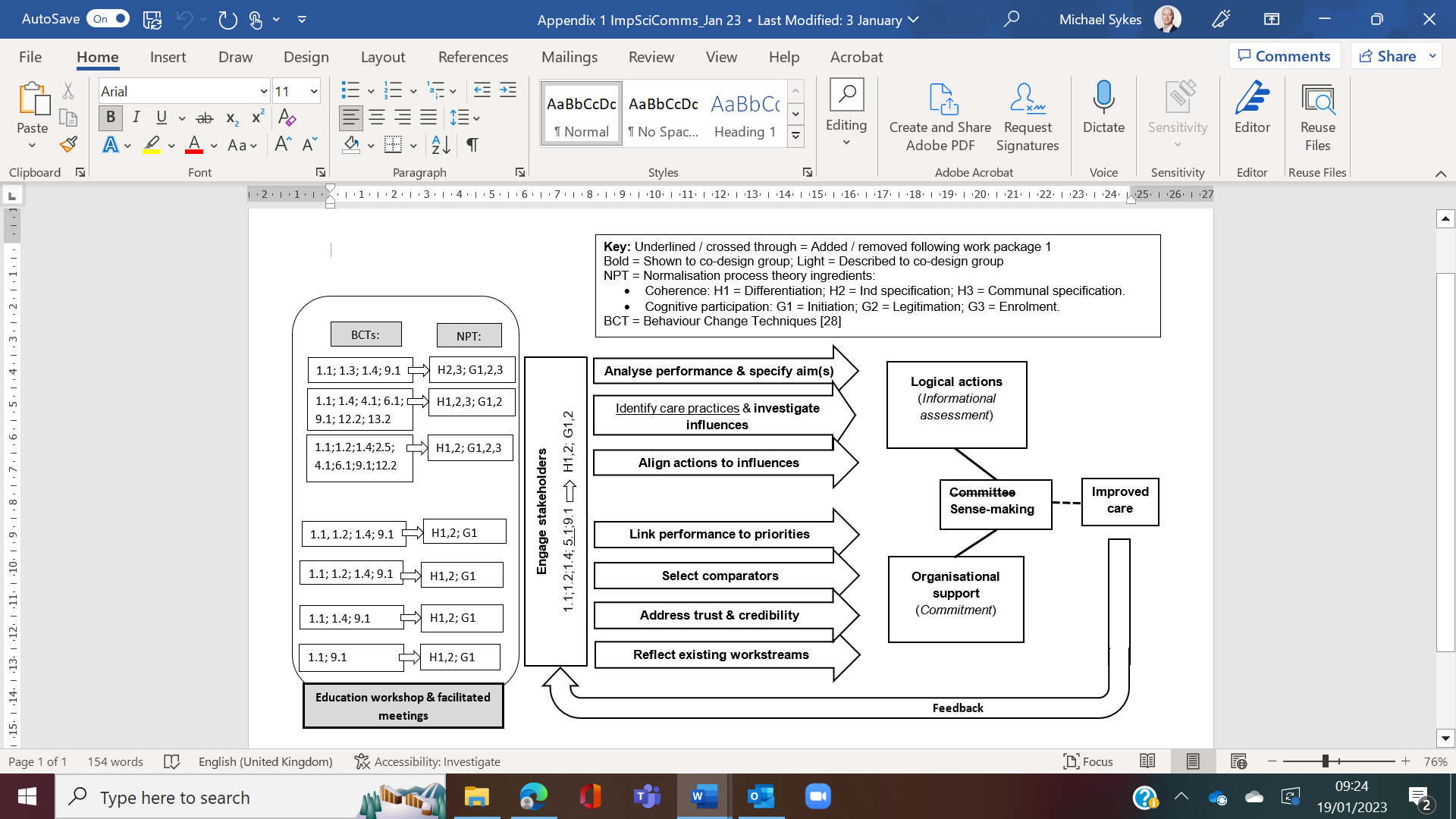


**Appendix 2:** TIDieR [27] and FRAME-IS [28] description of the post-adaptation QIC

**Template for intervention description and replication (TIDieR)**

(The key differences in TIDieR from the prior QIC underlined)

Brief name: Quality Improvement Collaborative

Why: To support the quality improvement capabilities of recipients of feedback from the national audit of dementia

What materials: PowerPoint slides

What procedures: To develop and implement improvement actions in response to the national audit of dementia through a structured process involving: Analysing performance and specifying target for improvement; Investigating barriers; Identifying actions; Link performance to priorities; Select comparators; Address trust and credibility; Reflect existing workstreams.

Who provided: Facilitator

To whom: Clinical leads from participating hospitals

How: Virtual interactive workshop and monthly virtual multisite facilitated calls

Where: Virtual (Microsoft Teams)

When: After audit feedback received. Three x 1-hour workshop and monthly calls (n=6)

Tailoring: Undertaken through supporting participants to tailor their response to local circumstances

**Additional Framework for Reporting Adaptations and Modifications to Evidence-based Implementation Strategies (FRAME-IS) sub-components:**

The evidence-based practice being implemented is identified by participants based upon analysis of clinical performance described by the national audit.

The reason for adaptation is to generate a positive affective response, be perceived as effective and appropriate and to lead to fidelity of receipt and enactment.

The level of adaptation is national, that is, to address differences between England and Ireland.

Who participated in adaptation? Seven clinical dementia leads from different clinical professions in three diverse hospitals within one Irish Hospital Group, and MS and ST from the research team.

**Appendix 3:** Interview topic guides (v30May22)**:**

Introductions, reminder of focus of the interview and confirmation that happy to proceed as per consent.

1. Thank you for meeting with me, please could you tell me about your role?
2. How long have you had it?
3. And how long have you worked in the health service?

I would like to ask you a few general questions about the approach to the INAD Quality Improvement Collaborative:

1. What did the Collaborative involve?
2. When I am talking about the Collaborative, I am talking about the workshops and the monthly calls. What was your overall experience of the workshops and monthly meetings?

(Seek to explore affective response, that is, how they feel about the three workshops and the monthly calls)

1. What do you think the workshops and meetings were trying to achieve? (intervention coherence)
2. Did they do that? (perceived effectiveness)
3. Did the Collaborative change what you were doing?

(If they don’t mention it, or if they need clarification, seek to explore whether it altered how they responded to the national audit results e.g. undertook different or additional actions to improve dementia or delirium care)

1. [If applicable]
   1. How did it do that? (To explore participant understanding of how intervention works)
   2. OR What might have changed what you were doing, in order to improve it?
2. What do you think about the time spent in the workshops and meetings?

(Explore both the perceived value / burden)

1. Did the Collaborative fit with your ways of working?

(To explore appropriateness of the intervention)

1. What have you done, as part of the project?
2. How did you develop your improvement projects?

Allow response, then explore more specifically, asking whether they:

1. Analysed performance and select a target
2. Used the exercise (below- shown to them at this point) to identify influences to select their actions
3. Sought to engage stakeholders
4. Discussed how the improvements links to existing goals or priorities
5. Discussed how your hospital performs compares to others
6. Discussed the source and quality of the national audit data
7. Discussed related existing workstreams, when discussing how to improve
8. Monitored whether you have improved
9. Would the Collaborative be applicable to other audits / processes in the hospital? (feasibility, suitability for use)
10. Did anything hinder/help you with your work in the quality improvement collaborative? If so, what? (To explore contextual influences)
11. This work only involved hospitals from one Hospital Group, what do you think would be the impact of involving hospitals from different Groups?

(NB. Also explore views on whether or not there should be the same number of hospitals in the collaborative in total)

1. Is there anything else you would like to say about the Quality Improvement Collaborative?

**Appendix 4:** Exemplar quotes from Work package 1

| **Summary of co-design group feedback**  The group described… | **Exemplar quotes from co-design group** | **Adaptation**  *(Stirman et al, 2013 Classification)* |
| --- | --- | --- |
| That there may be a lack of positional leader engagement with the audit (INAD) findings, and/or work to improve the care for people with dementia.  This lack of engagement may reduce both participants’ and stakeholders’ willingness to commit time to the QIC. | “The MDT are all very knowledgeable and they know what needs to happen, but there’s no provision or support from the top. There’s no governance from the top-down on it”  “It’s just not, well, a priority, you know, so it’s not seen as a priority within the hospital. There’s no sort of, big onus like, it’s not basically being published either, or in the news, there is no sort of like, I suppose, highlighting of it, so maybe that needs to happen.”  “we’re not all at that [organisation-level committee] table to discuss changes, we’re not all at the table to shout for dementia or shout for the opportunity that comes along. They only sort of filter down”  “The only opportunity I would have was to say to my Director of nursing, in the meetings I would have with her annually, to highlight what was needed from, you know, the audit”  “The last one I did with the assistant director of nursing” | National Dementia Office to engage senior leaders *(Integrating another approach into the intervention)*  Stakeholder engagement content to move earlier, so that it is considered before exploring influences upon performance, which requires stakeholder engagement. *(Re-ordering elements)*  Stakeholder engagement to be revisited in Workshop 2 (also add examples of the different stakeholders to engage; use ‘influences’ exercise to engage and gain perspectives upon influences) and again in Workshop 3 (here adding discussion of impacts from INAD performance that might relate to local priorities e.g. patient outcomes, costs, length of stay) *(Repeating elements; Extending).* Add a new engagement activity, so that participants consider the influence and interest of different stakeholders *(Adding elements)* |
| That the INAD report, and dementia care more broadly, may not be discussed at committees. These structural differences need to be reflected in the work to develop commitment. | Was there a hospital level action plan? “Not at all, no” “There’s no one representing care of the elderly at a committee level, at a directorate level, it’s not sexy, it’s not trendy” | Remove committee sense-making from logic model and identify other ways to engage stakeholders *(Substituting elements)* |
| That short sessions may fit better with participants working patterns. | “The other challenge is the time and the resources, especially when you have a small team like we have in [hospital], to give up an afternoon is a big ask.” | Split into three one-hour workshops, with post-workshop tasks (e.g. After workshop 1: Discuss INAD priorities with your colleagues. What does meeting these standards involve? Who re the stakeholders). To assist with sense-making, add reminder of previous content at the beginning of each workshop. *(Refining)*  Reduce duration by moving exercises outside the workshops and replacing them with group practice exercises.  *(Condensing)* |
| That virtual delivery would be appropriate. | “IT is the way forward”  “Virtual meetings are here to stay.” | Workshops are now often delivered virtually due to Covid-19 Pandemic and would reduce the relative travel time costs from splitting workshop into three sessions.  *(Contextual modification: Delivery channel)* |
| A preference to collaborate with other hospitals within their Hospital Group.  This was anticipated to create social opportunity to collaborate with hospitals that were similar, providing the opportunity for knowledge translation.  Working with hospitals within a Hospital Group was also anticipated to increase positional leader support and organisational commitment for change. | “I think it would be good if within the hospital group we could support each other”  ” “No, not really [there isn’t cross-group collaboration]” Why “The networks don’t exist, the group concept is relatively new. It’s a huge opportunity, isn’t it”  “We all know each other, but we don’t tend to tap in, and look for help from each other”  “We went to a presentation or group workshop…some of the other hospitals around the country from other groups were presenting their wonderful pathways they had worked out for dementia and delirium from the ED right way through the hospital and we just came out of it afterwards and there was no way we could take that template and make it work in our systems because they are so completely different”  “what I would say might come from it (collaborating across the group) is put a bit more pressure on management levels, to know that, well, the other hospitals within the group are getting involved with this, we need to be seen to be getting involved as well. I think it would bring about a bit more accountability I suppose, at that level.” “That’s a really good point, yeah” | Limit the QIC to one Hospital Group  *(Contextual modification: Level of delivery)* |
| That the clinical governance roles present within English hospitals that were studied, were not present in Ireland. These structural differences need to be reflected in the informational appraisal work. | The Quality Improvement Officer [name], he’s been newly appointed [at the hospital] I do get the impression that he’s spread pretty thinly because he seems to have multiple roles. I don’t think his sole focus is quality improvement, he’s also our patient liaison officer for complaints and he’s also seemingly got something to do with education... so I don’t feel like he’s someone who would have time to spare to tease through the nuances of something like this, he’s more of a fire fighter with problems that arise, for example, if we have a excess number of falls he gets involved rather than kind of…I don’t think its particular about seeking solutions, its more about responding to problems… We would seek him out, I’m not entirely sure that he would be actively following up with us. I’m not entirely sure that the result would land in his in box without being sent by us”  “I don’t think they would respond proactively, they would respond reactively. Not that they’d be disinterested in anything we had to say, but there would be very little forward action on that.” | Change participants so as not to seek clinical governance leaders for the workshops  *(Contextual modification: Personnel)* |
| **Workshop 1:** | | |
| That they currently select priorities for improvement based upon ease of action rather than impact upon meaningful outcomes. This may result in the selection of less effective actions, undermining the ability of the intervention to improve care. | “We checked what were easy wins…and we prioritised the easy wins first to get through those and then move forward” | To enable collective discussion of priorities with the greatest opportunity to improve patient outcomes, present INAD data for all participating sites, allow two minutes reflection, then ask what they would celebrate and what might be their priorities for improvement. *(Additional element)* |
| That there were differences of opinion about who would undertake a care practice, where and when. This reinforced the need to include content that supported teams to describe local practice. | - | To develop collective understanding of how to specify the target for improvement, add discussion to specify target for improvement using an example from the INAD standards (i.e. What does meeting the delirium screening standard involve, who does it, where, when and with what materials) as a step prior to exploring influences in workshop 2. *(Additional element)* |
| **Workshop 2:** | | |
| The importance of engaging clinical staff in order to gain buy-in for change. | “The only way you can change things is by knowledge and support on the ground.” | Add examples of the different stakeholders to engage. Use influences exercise to engage and gain perspectives upon influences *(Additional element)* |
| Differences in terminology in Ireland compared to England. Using Irish terminology would support understanding and credibility. | Changes such as remove ‘named nurse’, ‘safety huddle’ to ‘safety pause’ | Check terms used throughout  *(Refining)* |
| Selecting from a small range of potential implementation strategies. | “What I’ve noticed is, the only way you can actually improve care, and change, is by education and the national dementia champions course…they’re the only ways you can change is through knowledge and by support on the ground.” “I agree” | Add example of a completed logic model and group work to consider influences upon one standard. Standard selected based upon discussion in Workshop 1. *(Additional element)* |
| The potential for gaps in communicating agreed improvement actions. | “I have seen them [action plans] used, in different leadership and management programmes and that, but we don’t tend to, I suppose, do that.” “No definitely not.” “We just take the minutes of the discussion that actually happens.” “So the solutions weren’t assigned to different people to take action on them?” “No, there were one or two people that would have been nominated for jobs that might not necessarily have been asked, I’m not sure if he emailed them directly.” “That’s really good example of how things work in Ireland.” “I wasn’t at the meeting, and I didn’t get an email, I got a job alright (laughter), I heard about it on the grapevine” (laughter) “That’s a really good example.” “As an outsider who doesn’t go to the falls meeting, it doesn’t sound to me like a system that’s going to be effective.”  “I think we should still be drafting a report (to go to a committee), I just don’t have any confidence that they would be read…we can also use it to keep ourselves accountable, even if the higher-aboves aren’t, you know, reading it. It certainly has value.” | Add content about developing an action plan  *(Additional element)*  Revisit stakeholder analysis to include consideration of stakeholders to the actions *(Extending)* |
| That if they knew about existing actions, they may be able to amend them to improve INAD performance, but often they would not know about them.  Challenges with gaining organisational commitment to the intervention due to a lack of clear reporting lines. Engaging stakeholders may be more relational than structural. | “We actually got the delirium assessment put into the nursing Kardex. That was a huge win I suppose”  “I think I said this in the last group meeting as well, things don’t run through pathways, they don’t run through lines of management, if you want, the advice I was given when I started here was, if you want to do something, just do, start at a small level, bring as many people along as you can but there’s no point in sending a business plan to the hospital manager because it will never happen. There’s no clear lines of reporting, there’s no clear lines of approach in terms of implementing something…it’s about who you know, it’s about luck and its about being there at the right time. Writing an email or scheduling a conference (call), very little gets achieved.” | Add post-workshop work to consider:   - Stakeholders’ improvement goal(s) - stakeholders’ understanding of performance - strategies and workstreams related to the identified actions - local internal communication arrangements *(Additional element)* |
| **Workshop 3:** | | |
| That senior positional leaders may not prioritise INAD results. This might reduce organisational commitment for change. Linking INAD to other priorities including complaints, incidents, reputation, length of stay and/or other costs may increase positional leader engagement and organisational commitment. | “It’s just trying to get resources and specific people put in…we have done business cases in dementia and all that, but it’s just not one of the priorities. It’s not seen as a priority in the hospital. There’s no big onus. It’s not been published either, you know, or in the news. We’re not being touched, so there’s no sort of like, I suppose, like highlights saying [hospital] did so bad. Maybe that needs to happen.”  What would be persuasive in those conversations to gain Director of Nursing commitment? “I think it would be scenarios where things have gone wrong, for example, patient complaints or family complaints. I don’t think they would respond proactively, I think they would respond reactively. Not that they’d be disinterested in anything we had to say, but there would be very little forward action on that.”  “The falls committee would have fallen to the wayside as well, until [incident reports] started happening, until falls became a big issue within the hospital. All of a sudden, general management have taken an interest because it’s brought the ‘higher-ups’…dementia gets pushed aside because there aren’t major issues, funding, effects from the dementia patients…falls is the perfect example, until general management gets involved, change won’t happen”  “I think reputation probably is important, but it’s the big, the big media leaks and things that really impact, and like x number of people died from heart attacks who attended the hospital. It’s hard to think what data might be in INAD that would be in any way interesting to the media, would be hyped up and publicised. Certainly reduced care costs, and I think we’d need to put a lot of thought into how any work like this would reduce length of stay. The cynical part of me would say that they don’t really care about patient experience, and the CQC (Care Quality Commission) equivalent here is not as scary as the CQC…I don’t think they wield as much power and I don’t think the inspections carry as much weight. as they do in the NHS. Again that’s something that I’d be interested to pick the GMs brains about. So I think that the priorities for the hospital, we could definitely expand on that, and I think I would need to understand that a bit better.”  “Even a corridor conversation with a GM [general manager] might enlighten us as to what is important to them.”  “If we match it to length of stay and to cost, then it will become a hot topic. That’s the only way I think. Or staffing, HCAs, needing 1 to 1 care.” | Add discussion of impacts from INAD performance in improvement goal that might relate to local priorities e.g. patient outcomes, costs, length of stay *(Additional element)* |
