# Supplementary material for: Adapting a quality improvement collaborative to a new national context: a co-design and feasibility study to improve dementia care in Ireland
Source: BMC Health Serv Res. 2023 Oct 4;23:1056. doi: 10.1186/s12913-023-10019-3 (PMC10548569; doi:10.1186/s12913-023-10019-3)

**Supplementary material:** The evolution of data categories

Version 1:

Category

- Collaborate
- Facilitation
- Keeping on track
- Commitment
- Engagement
- Reach
- Existing workstreams
- Monitor change
- Sustain
- Affective response
- Motivation
- Coherence
- Appropriate
- Cost/Burden
- Perceived effectiveness
- Take action
- Prioritise
- Influences

Version 2:

1. **Inside** the QIC intervention: Things about the QIC itself, how to do it, was it good, what was good
   - - Collaboration
     - Facilitation
     - Set structure
     - Motivation
     - Appropriate
     - Cost/burden
     - Perceived effectiveness
     - Extend collaboration
2. **Outside** of the QIC: Things resultant from the QIC, effected by the QIC, stemming from it
   - - Commitment
     - Engagement
     - Existing workstreams
     - Monitor change
     - Sustain
     - Reach
     - Iinterpretation
     - Take action
     - Prioritise
     - Influence
     - Discuss data quality

Early model drawing associations between categories:


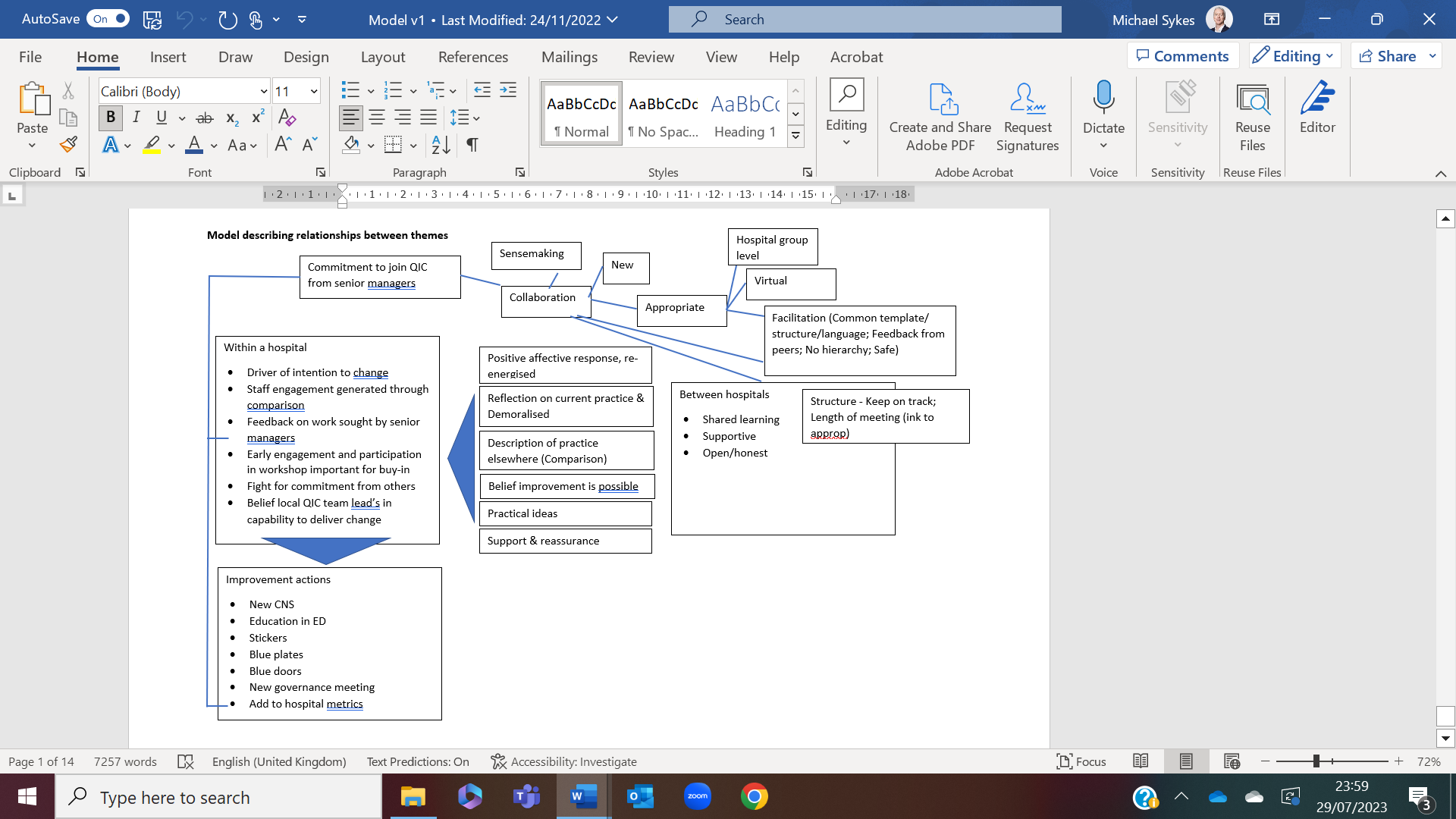

Supplement: Supplementary file 2 — Additional file 2: Supplementary material. The evolution of data categories. [file 12913_2023_10019_MOESM2_ESM.docx]
